# Supplementary material for: Under-five mortality before and after implementation of the Liberia National Community Health Assistant (NCHA) program: A study protocol
Source: PLoS One. 2024 Mar 1;19(3):e0272172. doi: 10.1371/journal.pone.0272172 (PMC10906894; doi:10.1371/journal.pone.0272172)
Supplement: S1 Table — (DOCX) [file pone.0272172.s002.docx]

S1 Table: Descriptive statistics remote Grand Bassa

| Remote Grand Bassa County | | |
| --- | --- | --- |
| *N* Communities | 1,733 | |
| *N* Households | 23,702 | |
| *N* Health facilities | 34 | |
| Average distance (remote community) | 13.5 km | |
|  |  |  |
| Households | | |
| 1^st^ and 2^nd^ rural wealth index quintiles (DHS 2013) | 70% | |
| No toilet facility | 88% | |
| Access to an improved non-shared toilet facility | 2% | |
| Access to an improved water source | 22% | |
|  |  |  |
| Women | | |
| Attended some school | 34% | |
| Completed primary school | 11% | |
| Married or cohabitating | 78% | |
| Bassa language | 60% | |
| Antenatal care visits (> 3) | 63% | |
| Postnatal care visits within 48 hours | 37% | |
| In facility delivery | 53% | |
|  |  |  |
| Children under 5 | | |
| Disease | Prevalence | Treated by a qualified provider |
| Diarrhea | 50% | 75% |
| Fever | 54% | 62% |
| ARI | 28% | 67% |
|  |  | |
| Vaccination | Coverage | |
| BCG, Penta, OPV and Measles | 21% | |
| Note: Data covers all communities more than 5 km from the nearest health facility based on 2018 Last Mile Health’s Grand Bassa household survey. ARI = acute respiratory infections, BCG = Bacillus Calmette–Guérin, OPV = Oral polio vaccine. | | |
